# Evans’ Index and Frontal and Occipital Horn Ratio in Correlation to Volumetric Data of Lateral Ventricles in a Population-based MRI Study

**DOI:** 10.1007/s00062-026-01665-1

**Published:** 2026-05-06

**Authors:** Daniel Cantré, Robin Bülow, Eiko Rathmann, Jörg Baldauf, Felix Streckenbach, Sönke Langner

**Affiliations:** 1 Institute and Policlinic of Radiology, Paediatric Radiology and Neuroradiology, University Medical Centre Rostock, Rostock, Germany; 2 Institute of Diagnostic Radiology and Neuroradiology, University Medicine Greifswald, Greifswald, Germany; 3 Department of Neurosurgery, University Medicine Greifswald, Greifswald, Germany

**Keywords:** Evans’ Index, Frontal and Occipital Horn Ratio, Hydrocephalus, MRI volumetry

## Abstract

**Purpose:**

Evans’ Index (EI) and Frontal and Occipital Horn Ratio (FOHR) are routinely used in screening for enlargement of the ventricular system. However, both lack epidemiological definition of normal values in adults. In a representative, randomized sample of adult normal population in Germany we studied the correlation of lateral ventricle volumes with EI and FOHR to evaluate the performance of pre-established cutoff values of EI > 0.3 and FOHR > 0.37, and to introduce age- and sex-specific thresholds, which for FOHR do not yet exist in adults.

**Methods:**

In a population-based sample of 3058 subjects of the “Study of Health in Pomerania” (SHIP), aged 21–90 years, brain imaging with T1 weighted MPRAGE with 1 mm isotropic voxel-size was obtained. We semiautomatically determined the volumes of the lateral ventricles and manually performed measurements to calculate EI and FOHR for every individual. Besides descriptive statistics, ROC analyses were carried out to determine test performance of both parameters. Negative and positive predictive values were derived from contingency analyses.

**Results:**

Average values were 0.257 ± 0.025 for EI and 0.363 ± 0.024 for FOHR, with sex- and age-depending differences. Using the established cut-off value of EI > 0.3, this index achieved high specificity of 97.1% and moderate sensitivity of 55.3% for diagnosing ventricular enlargement. Using a threshold of > 0.4, FOHR achieved a specificity of 93.4%, and higher sensitivity of 81.4%. FOHR showed stronger correlation with actual lateral ventricle volumes, as compared to EI (r_s FOHR_ = 0.741 vs. r_s EI_ = 0.656; *p* < 0.001).

**Conclusion:**

For the first time, EI and FOHR were evaluated in a reasonable sized, randomized, representative sample of normal adult population in Germany, and were confirmed as useful screening tools in the diagnosis of ventricular enlargement. We introduce a cut-off value of > 0.4 for FOHR in adults, with emphasis on specific caveats.

## Introduction

The cerebral ventricular system is an important anatomic part of the human brain and routinely evaluated by medical imaging [1]. Interpretation of ventricular size is a daily task for neuroradiologists, not only to rule out hydrocephalus, but also as a sign of brain atrophy in the differentiation of neurodegenerative diseases [2]. However, valid data on physiological age-dependent changes of ventricular size and volume is scarce [3, 4]. The majority of publications focus on pathological aspects of ventricular size in retrospective studies of diseased or at least hospitalized subjects with small sample sizes [5, 6], yielding the risk for sampling error, because healthy and younger individuals rarely undergo brain-imaging. Therefore, results are usually not representative of the normal population. However, knowledge of age-related normal values is essential for valid differentiation of ventricular pathology from changes that lie within normal range of an individual’s age. While some population-based data of specific age groups became available over the last decade, a comprehensive population-based evaluation of all adult age groups in central Europe is still lacking [7, 8]. In the course of many neurological and psychiatric diseases, for example Alzheimer’s disease [9], atypical parkinsonian syndromes [10], multiple sclerosis [11], schizophrenia [12] or bipolar affective disorder [13], brain atrophy can occur, leading to secondary dilation of the ventricular system. On the other hand, it is recognized that there is age-related physiological brain atrophy, that may also lead to ventricular enlargement [14]. With established age-related normal values, brain imaging might provide even better information in the work-up of neurodegenerative or psychiatric diseases.

In 1942 W. A. Evans described a simplified method to estimate ventricular width by measuring the diameter between the lateral borders of both frontal horns of the lateral ventricles and compare it to the maximum inner skull diameter. The resulting quotient is thus called the Evans’ Index (EI) [15, 16]. An EI greater than 0.3 is since regarded to indicate ventricular enlargement and can be found as a cut-off value in international guidelines to this day [17]. Meanwhile, further methods have been developed, e.g. by Huckmann et al. [18], the “cella-media-index” [19, 20] or the “ventricle-brain-ratio” [21]. In 1998, O’Hayon et al. proposed the Frontal and Occipital Horn Ratio (FOHR) as a specific indicator for dilation of the ventricular system in children [22]. Compared to EI, FOHR showed higher correlation to the ventricular volume (VV) by adding the diameter between the lateral borders of the occipital horns in the equation. O’Hayon et al. described a FOHR of 0.37 as a normal value for pediatric patients. However, an absolute cut-off value for elevated ventricular system volume has not yet been identified. In 2003 Jamous et al. described the Frontal and Occiptal Horn Width Ratio (FOHWR) [23]. This index uses the individual diameters of frontal and occipital horns of the lateral ventricles of either side. Compared to both EI and FOHR, correlation of FOHWR to actual ventricular volume was even higher but needs substantially more measurements performed.

The aims of this study were to measure the volumes of the lateral ventricles in a large, randomized sample of normal population in Germany, and to assess correlation between ventricular volumes and the indices EI and FOHR. Further, we wanted to assess test performance including predictive values of both EI and FOHR, and to establish a cut-off value for FOHR in an adult population for the first time.

## Materials and Methods

### Probands

All probands were part of the “Study of Health in Pomerania” (SHIP) [24]. The study was Performed according to the Declaration of Helsinki in its present form; Ethical approval was given by the local Review Board. Written and oral informed consent was obtained from all participants prior to inclusion into the SHIP-study, which was performed by random sampling, using official data of resident’s registration offices in consideration of sex and age. All probands who received brain MRI imaging within the SHIP-study in the SHIP-2- and SHIP-TREND-cohorts were included in our analysis [25]. Exclusion criteria were missing transversal T1 weighted MPRAGE MRI-sequence of the brain, evident intracranial pathology other than isolated ventricular enlargement, e.g. tumors, post-hemorrhagic or postischemic defects, and inadequate quality of imaging data. At the time of evaluation, 2988 datasets of probands could be included. Gender ratio was 52.7 % females (*n* = 1576) and 47.3 % males (*n* = 1412). Mean age for the total cohort was 52.7 ± 13.6 years (range 21 to 90 years). For further evaluation, subjects were divided into seven age groups (AG) 2 to 8 (Table 1).

**Table 1:** Definition of age groups (AG) 2 to 8 and distribution of probands.

AG (years of age)	Probands (%)	Women (% of AG)	Men (% of AG)
2 (20–29)	151 (5.1)	66 (43.7)	85 (56.3)
3 (30–39)	379 (12.7)	202 (53.3)	177 (46.7)
4 (40–49)	730 (24.4)	394 (54.0)	336 (46.0)
5 (50–59)	724 (24.2)	383 (52.9)	341 (47.1)
6 (60–69)	650 (21.8)	359 (55.2)	291 (50.8)
7 (70–79)	317 (10.6)	156 (49.2)	161 (50.8)
8 (≥ 80)	37 (1.2)	16 (43.2)	21 (56.8)
All	2988 (100)	1576 (52.7)	1412 (47.3)

Note: *AG* age group

### MRI Protocol

MRI was performed at a 1.5 T whole body MR system (Magnetom Avanto, Siemens Healthineers, Erlangen, Germany). The full scanning protocol is published elsewhere [26]. For the presented analysis, axial T1-weighted MPRAGE sequences with 1 mm isotropic voxel size (TR 1900 ms, TE 3.4 ms, flip angle = 15°; DICOM file format) of the entire brain were used.

### Manual Measurements and Calculation of EI and FOHR

**Fig. 1:**
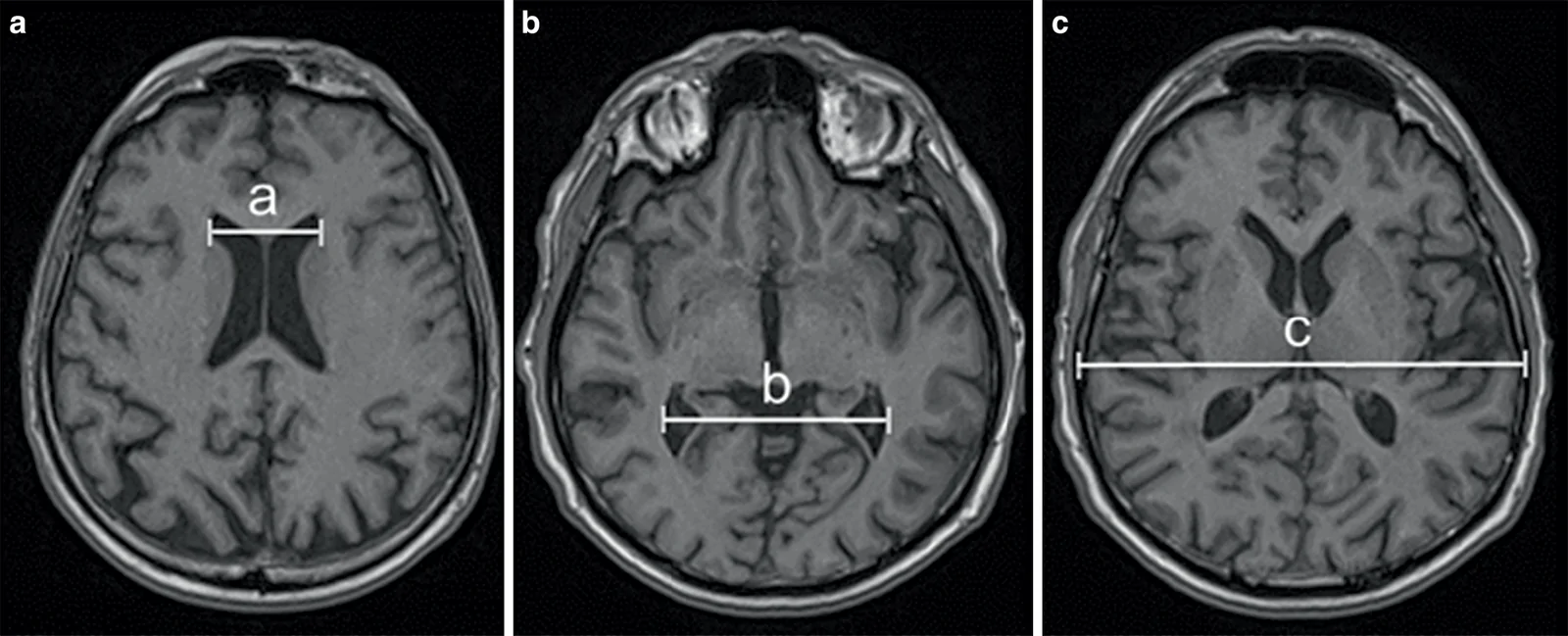
Manual measurements performed for the calculation of Evans’ Index (EI) and Frontal and Occipital Horn Ratio (FOHR). Maximum distance between the lateral borders of the frontal horns (**a**), and the occipital horns (**b**) of the lateral ventricles. Maximum inner skull diameter (**c**). Each item must be measured strictly horizontal on the individual axial slice where it reaches its maximum. Ratios were calculated using the formulas: *EI* a/c; *FOHR* (a + b)/2c

The Evans’ Index (EI) is defined as the quotient of the widest diameter between the lateral borders of both frontal horns of the lateral ventricles and the widest inner skull diameter [15, 16]. For the Frontal and Occipital Horn Ratio (FOHR), the sum of the maximal diameters of both frontal and both occipital horns of the lateral ventricles are summarised and then divided by two times the greatest inner skull diameter [22]. The manual measurements were performed strictly horizontal on axial 1 mm slices of the T1-weighted MPRAGE using OsiriX® DICOM viewer (Version 3.9, [27]) with standardized windowing parameters and a zoom factor of 385 %. Each measurement was performed on the one slice with the maximum diameter for the individual item (Fig. 1). For every participant, the whole axial T1w MPRAGE image stack was visually screened by the readers, and the axial slices with the individual maximum diameters were chosen by the discretion of the readers. A random dataset of *n* = 100 of all cases was re-evaluated by a board certified neuroradiologist with more than 10 years of experience in neuroimaging to determine interrater reliability (IRR).

### Lateral Ventricular Volume

Segmentation and volumetric analysis of the ventricular system was performed semiautomatically, using the analysis tool FAST (“FMRIB’s automated segmentation tool”; version 4.1) from the FMRIB software library (FSL; version 4.1.8) [28–30]. MRI data were segmented into grey matter (GM), white matter (WM) and cerebrospinal fluid (CSF) [31–33]. First, a so called “skull stripping” was performed with the FSL-tool BET (Brain Extraction Tool; version 2.1), using a cut-off value of 0.3 [34], followed by spatial normalization with the MNI-template (Montreal Neurological Institute; [35]) by the FLS-Tools FLIRT and FNIRT (FMRIB’s Linear Image Registration Tool; version 5.5, FMRIB’s Non Linear Image Registration Tool; build 417) [36–38]. The normalized data were then sent to the FAST tool, which performed a segmentation of the three given textures in original spatial resolution (voxel volume 1 × 1 × 1 mm), and voxel-based calculation of the volumes of GM, WM and CSF. For determination of the volumes of lateral ventricles only, automatic lateral ventricle delineation (ALVIN) was used, a method developed by Kempton et al. using data of 275 healthy subjects aged 18–94 [39]. ALVIN provides a mask including frontal, temporal and occipital horns of both lateral ventricles that can be applied to the normalized data of CSF volume. This mask had to be upscaled to a voxel size of 1 × 1 × 1 mm to adjust for the original spatial resolution of our normalized data, segmented with FSL. The result was then cut in mid-sagittal plane to obtain symmetrical masks for each hemisphere, to calculate the individual volumes of left and right lateral ventricles. Processing time for each dataset was 10 min.

**Fig. 2:**
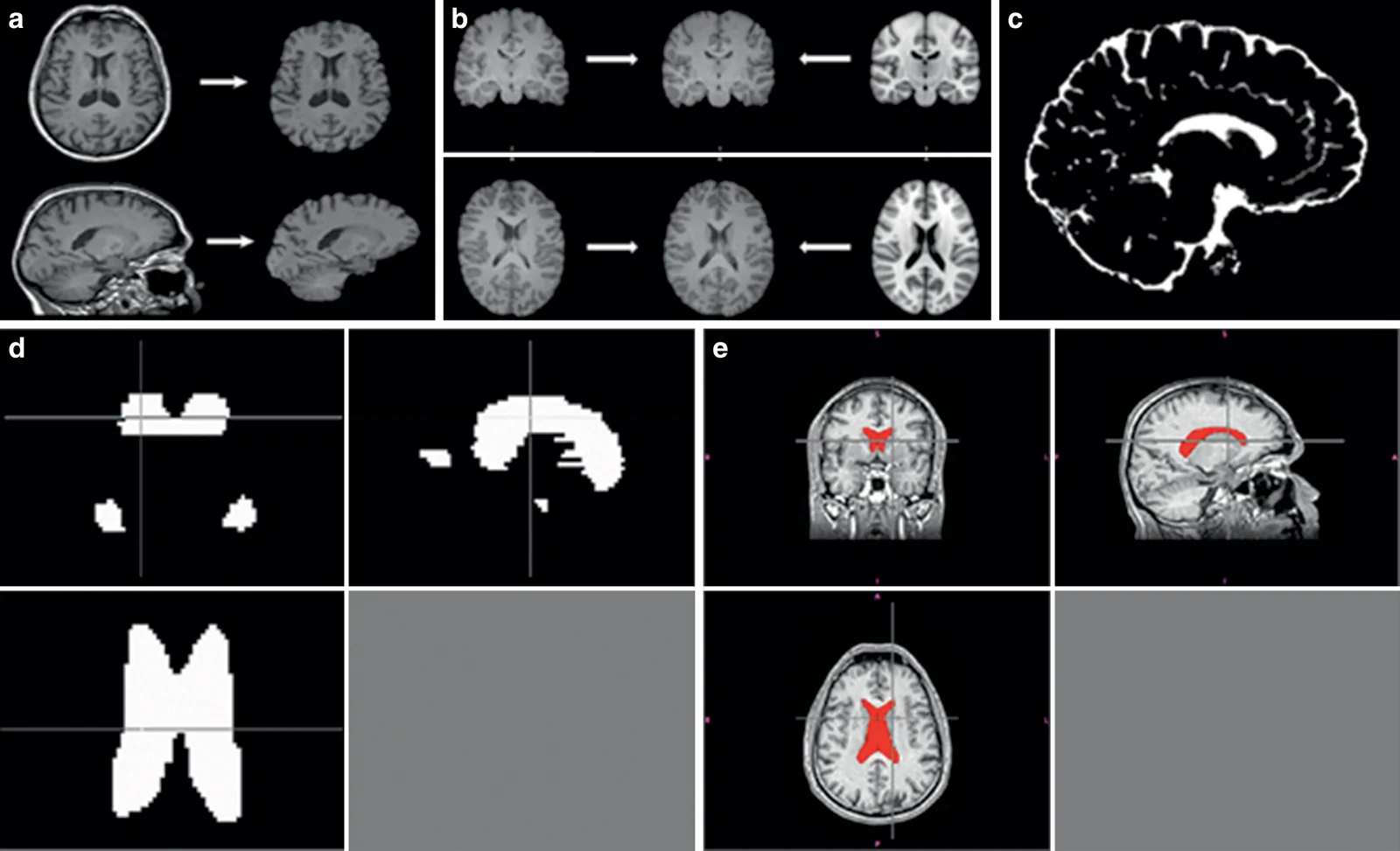
Workflow of semiautomatic volumetry of the lateral ventricles. “Scull stripping” (**a**) and spatial normalization to the NMI template (**b**), was followed by automatic segmentation of white and gray matter (not shown), as well as cerebrospinal fluid (**c**). Semiautomatic determination of the lateral ventricle volumes using ALVIN (**d**). Visual quality control (**e**) through overlay of the segmentation results on the T1-weihgted MRI source images

After semiautomatic volumetry of the ventricular system with ALVIN, visual control of all results was done using the FLSView tool (Version 3.1.8), to confirm exact correlation of the created masks with actual borders of the ventricular system as pictured in the T1 weighted source images (Fig. 2). According to the quality of subjective visual correlation, datasets were divided into three categories: in category 0 there was excellent visual correlation; category 1 covered datasets with good correlation with only minimal overlap; and category 2 was assigned to datasets with inadequate visual correlation, where gross overlap was seen. All Datasets of category 2 were excluded from further evaluation. IRR of visual correlation and assignment to the given categories was also determined by re-evaluation of *n* = 100 random datasets by an experienced neuroradiologist.

### Statistics

IBM SPSS Statistics (Version 20.0 and above) was used for statistical analysis. For calculation of correlation and interrater reliability (IRR) by means of quality control, Spearman’s rank correlation coefficient (r_s_) was determined. Normal distribution of data was tested by Kolmogorow-Smirnow-Test, and for evaluation of sex-dependent differences we used t‑Test. LVV enlargement was determined as volumes above the 95th percentile, allowing for robust analysis of age- and sex-dependent subgroups independent the distribution of the data. To find optimal threshold values for EI and FOHR, we performed ROC-analyses and calculated the Youden Indices (YI). Comparison of the areas under the ROC-curves (AUROC) of EI and FOHR was carried out using the DeLong test. Negative predictive values (NPV) and positive predictive values (PPV) were determined by contingency analysis, applying different cutoff-values for both parameters: a) best performing cutoff-value as determined by the highest YI, b) pre-established cutoff-values (EI > 0.3 and FOHR > 0.37), c) cutoff-value where 50 % PPV was reached, and d) for FOHR a balanced cutoff-value derived from the whole cohort (FOHR > 0.4) for comparison in age- and sex-dependent subgroups. *P*-values with *p* < 0.05 were regarded as statistically significant.

## Results

Measurement results of skull diameters, frontal horn diameters and occipital horn diameters, as well as the derived calculated EI and FOHR, and the results of semiautomatic lateral ventricle volumetry (LVV) including the 95th percentile thresholds of LVV are presented in Table 2. Mean total LVV of the complete cohort was 22.8 ± 11.0 ml (mean ± SD, range 4.9 to 69.7 ml). In the female subgroup, mean total LVV was 20.0 ± 9.5 ml (range 4.8 to 69.0 ml) and in the male subgroup 26.0 ± 11.7 ml (range 5.9 to 69.7 ml). In both sexes, mean lateral ventricular volumes grew with age, with a mean growth of 21.9 ml (+153.7 %) in females and 25.8 ml (+134.9 %) in males between age group (AG) 2 to AG 8. When tested for normal distribution, LVV revealed positive skewness in age- and sex-dependent subgroups in AG 2 to AG 7 (*p* < 0.001). Only in AG 8 was LVV normally distributed. For the purpose of this study, we used a volume-threshold of LVV values over the 95th percentile to define lateral ventricular enlargement [5]. IRR-values were r_s_ = 0.897 for maximum inner skull diameter, r_s_ = 0.942 for maximum diameter of the frontal horns of the lateral ventricles, r_s_ = 0.760 for maximum diameter of the occipital horns of the lateral ventricles, r_s_ = 0.919 for EI and r_s_ = 0.794 for FOHR. Level of significance was *p* < 0.001 for all coefficients.

**Table 2:** Measurement results in Millimeters, Volumetry results in Milliliters and calculated parameters of all probands and according to the different age groups and gender presented as mean with standard deviation (SD); 95th percentile of LVV values represent the age-group- and sex-specific thresholds for enlargement of LVV.

Age Group	Sex	Count	Manual measurements [mm]						Calculated Parameters				Volumetry [ml]		
			Skull Diameter		FH Diameter		OH Diameter		EI		FOHR		LVV		
			Mean	SD	Mean	SD	Mean	SD	Mean	SD	Mean	SD	Mean	SD	95th percentile
AG 2	Male	85	135.43	4.87	35.08	3.23	61.85	3.20	0.259	0.023	0.358	0.017	19.09	8.78	33.24
	Female	66	131.59	4.91	33.12	3.16	57.64	3.50	0.252	0.023	0.345	0.018	14.26	6.90	27.73
	Total	151	133.75	5.23	34.23	3.34	60.01	3.93	0.256	0.023	0.352	0.019	16.98	8.34	33.18
AG 3	Male	177	136.92	5.22	35.42	3.08	62.83	4.07	0.259	0.022	0.359	0.018	19.40	7.55	34.07
	Female	202	132.35	4.77	32.58	2.83	58.97	3.69	0.246	0.021	0.346	0.015	15.38	6.20	26.97
	Total	379	134.48	5.48	33.91	3.27	60.77	4.32	0.252	0.022	0.352	0.018	17.26	7.14	31.49
AG 4	Male	336	137.95	5.18	35.75	3.45	64.52	4.50	0.259	0.023	0.363	0.020	21.45	9.17	41.33
	Female	394	133.82	5.10	33.31	3.31	60.47	4.08	0.249	0.023	0.350	0.018	17.52	7.54	31.72
	Total	730	135.72	5.53	34.43	3.59	62.33	4.73	0.254	0.023	0.356	0.020	19.33	8.55	35.78
AG 5	Male	341	140.06	5.24	36.60	3.74	66.69	4.64	0.261	0.025	0.369	0,021	24.68	9.83	43.49
	Female	383	135.45	4.94	33.60	3.15	62.40	5.62	0.248	0.022	0.354	0.023	18.53	7.91	33.74
	Total	724	137.62	5.58	35.01	3.75	64.42	5.60	0.254	0.025	0.361	0.023	21.43	9.38	39.03
AG 6	Male	291	139.93	5.36	37.37	3.73	68.53	4.51	0.267	0.026	0.378	0.021	30.67	10.39	51.44
	Female	359	136.37	5.05	34.79	3.50	64.71	4.09	0.255	0.024	0.365	0.020	23.45	9.70	43.20
	Total	650	137.97	5.48	35.94	3.83	66.42	4.68	0.261	0.026	0.371	0.021	26.68	10.63	48.13
AG 7	Male	161	141.73	5.31	39.02	4.30	71.50	4.55	0.275	0.029	0.390	0.023	38.35	12.26	58.45
	Female	156	136.74	5.33	35.61	3.94	66.32	4.90	0.260	0.027	0.373	0.023	28.36	11.51	51.76
	Total	317	139.27	5.87	37.35	4.46	68.95	5.39	0.268	0.029	0.381	0.024	33.43	12.89	56.52
AG 8	Male	21	140.44	4.70	41.13	4.14	73.54	4.90	0.293	0.026	0.408	0.019	44.85	10.83	61.16
	Female	16	137.75	4.83	36.57	3.78	70.71	3.30	0.265	0.024	0.389	0.020	36.17	9.36	55.16
	Total	37	139.28	4.88	39.16	4.55	72.31	4.46	0.281	0.029	0.400	0.021	41.10	10.98	61.16
AG 2–4	Male	598	137.29	5.22	35.56	3.32	63.64	4.33	0.259	0.023	0.361	0.019	20.51	8.72	38.15
	Female	662	133.15	5.05	33.07	3.17	59.73	4.02	0.248	0.022	0.348	0.017	16.54	7.19	29.57
	Total	1260	135.11	5.53	34.25	3.47	61.58	4.61	0.253	0.023	0.355	0.019	18.42	8.19	33.98
AG 5–8	Male	814	140.35	5.32	37.47	4.00	68.48	4.97	0.267	0.027	0.377	0.023	30.05	11.96	53.82
	Female	914	136.07	5.07	34.46	3.53	64.12	5.20	0.253	0.024	0.362	0.023	22.45	10.15	42.54
	Total	1728	138.09	5.61	35.88	4.05	66.17	5.54	0.260	0.027	0.369	0.024	26.03	11.67	49.84
AG 2–8	Male	1412	139.06	5.49	36.66	3.85	66.43	5.28	0.264	0.026	0.371	0.023	26.01	11.69	50.19
	Female	1576	134.85	5.26	33.88	3.45	62.27	5.21	0.251	0.024	0.356	0.022	19.97	9.48	38.08
	Total	2988	136.84	5.77	35.19	3.90	64.24	5.64	0.257	0.025	0.363	0.024	22.82	11.00	45.26

Note: *AG* age group; *SD* standard deviation; *FH* frontal horn; *OH* occipital horn; *EI* Evans’ Index; *FOHR* Frontal and Occipital Horn Ratio; *LVV* lateral ventricle volume

Mean EI for the whole cohort was 0.257 ± 0.025. Females had a mean EI of 0.251 ± 0.024 and males of 0.264 ± 0.026, respectively. Age related changes of EI were comparable to the changes in actual LVV, showing only a slight slope in AG 2–4, and a marked elevation for the AG 5–8 over 50 years of age. Similarly, in the younger age groups AG 2–4 (*n* = 1260) there was no correlation (r_s_ = 0.008; *p* = 0.78), whereas in AG 5–8 (*n* = 1728) there was significant correlation (r_s_ = 0.208; *p* < 0.001) between age and EI. For EI and total LVV, a correlation coefficient of r_s_ = 0.656 (females r_s_ = 0.622; males r_s_ = 0.643) was calculated, with levels of significance of *p* < 0.001 for all coefficients. Differences in the correlation coefficients for EI and LVV between AG 2–4 and AG 5–8 were small, but significant (r_s_ = 0.619 vs. r_s_ = 0.687; *p* < 0.001).

Mean FOHR for the whole cohort was 0.363 ± 0.024. Females showed a mean FOHR of 0.356 ± 0.022 and males of 0.371 ± 0.023, respectively. For FOHR and total LVV, a correlation coefficient of r_s_ = 0.741 was calculated (women r_s_ = 0.707, men r_s_ = 0.735), with levels of significance of *p* < 0.001 for all coefficients. FOHR showed stronger correlation with actual lateral ventricle volumes, as compared to EI (r_s FOHR_ = 0.741 vs. r_s EI_ = 0.656; *p* < 0.001).

Because of the observed age- and sex-dependent differences, ROC-analyses including determination of Youden Indices (YI) as well as contingency analyses were carried out for the whole cohort, as well as for two age-defined subgroups (AG 2–4 and AG 5–8) further divided by sex. The results for EI are presented in Table 3 and the results for FOHR are presented in Table 4, giving an overview of the different ratio cutoff values and their respective test criteria, i.e. sensitivity, specificity, negative predictive value (NPV) and positive predictive value (PPV).

**Table 3:** Results of test performance analysis by means of ROC-analysis and contingency analysis of Evans’ Index (EI). The different EI cutoffs were applied to each of three different AG-ranges, both gender-wise and combined. EI cutoffs chosen were the one with the highest Youden Index (YI), the pre-established value of > 0.3 and the one next to and over a PPV of 50%, if not already achieved by one of the former.

Age Group	Sex	EI ± SD	AUROC (95 % CI)	EI Cutoff Category	EI Cutoff Value	Sensitivity [%]	Specificity [%]	NPV [%]	PPV [%]
AG 2–8	Male	0.264 ± 0.026	0.927 (0.902–0.952)	Highest YI	0.280	91.4	78.5	99.4	18.1
				Pre-established	0.300	65.7	94.6	98.1	38.7
				50 % PPV	0.311	45.7	97.8	97.2	51.6
	Female	0.251 ± 0.024	0.919 (0.888–0.950)	Highest YI	0.273	83.3	87.0	99.0	25.1
				Pre-established	0.300	33.3	98.6	96.6	55.3
				50 % PPV	0.295	43.6	97.8	97.1	50.7
	Total	0.257 ± 0.025	0.930 (0.912–0.947)	Highest YI	0.279	85.3	85.1	99.1	23.2
				Pre-established	0.300	55.3	97.1	97.6	50.0
AG 2–4	Male	0.259 ± 0.023	0.961 (0.934–0.987)	Highest YI	0.281	93.1	87.2	99.6	27.0
				Pre-established	0.300	58.6	98.2	97.9	63.0
				50 % PPV	0.295	75.9	96.7	98.7	53.7
	Female	0.248 ± 0.022	0.887 (0.835–0.939)	Highest YI	0.261	76.5	78.7	98.4	16.3
				Pre-established	0.300	17.6	99.5	95.7	68.7
				50 % PPV	0.298	17.6	99.2	95.7	54.5
	Total	0.253 ± 0.023	0.944 (0.915–0.972)	Highest YI	0.273	92.1	85.1	99.5	24.6
				Pre-established	0.300	46.0	99.4	97.2	80.6
				50 % PPV	0.290	66.7	96.5	98.2	50.0
AG 5–8	Male	0.267 ± 0.027	0.914 (0.876–0.952)	Highest YI	0.290	85.0	84.8	99.1	22.4
				Pre-established	0.300	65.0	91.5	98.1	28.3
				50 % PPV	0.320	45.3	97.7	97.2	51.3
	Female	0.253 ± 0.024	0.944 (0.920–0.968)	Highest YI	0.273	91.1	84.1	99.5	22.9
				Pre-established	0.300	42.2	97.8	97.0	50.0
	Total	0.260 ± 0.027	0.939 (0.920–0.957)	Highest YI	0.280	91.9	82.6	99.5	21.7
				Pre-established	0.300	62.8	95.4	98.0	41.5
				50 % PPV	0.309	45.3	97.7	97.2	51.3

**Table 4:** Results of test performance analysis by means of ROC-analysis and contingency analysis of Frontal and Occipital Horn Ratio (FOHR). The different FOHR cutoffs were applied to each of three different AG-ranges, both gender-wise and combined. FOHR cutoffs chosen were the one with the highest Youden Index (YI), the pre-established value of > 0.37 (*initially validated for children) and the one next to and over a PPV of 50%, if not already achieved by one of the former. Additionally, in the age-derived subgroup analyses, the cutoff of > 0.4, achieving a PPV of 50% in the whole sample was evaluated.

Age Group	Sex	FOHR ± SD	AUROC (95 % CI)	FOHR Cutoff Category	FOHR Cutoff Value	Sensitivity [%]	Specificity [%]	NPV [%]	PPV [%]
AG 2–8	Male	0.371 ± 0.023	0.951 (0.902–0.952)	Highest YI	0.395	88.6	89.6	99.3	30.7
				Pre-established*	0.370	100	55.6	100	10.5
				50 % PPV	0.412	57.1	97.3	97.8	52.6
	Female	0.356 ± 0.022	0.964 (0.948–0.979)	Highest YI	0.374	94.9	86.5	99.7	26.8
				Pre-established*	0.370	96.2	80.8	99.8	20.7
				50 % PPV	0.390	57.1	97.3	97.8	52.6
	Total	0.363 ± 0.024	0.964 (0.955–0.973)	Highest YI	0.382	96.0	85.3	99.8	25.7
				Pre-established*	0.370	100	69.0	100	14.6
				50 % PPV	0.400	70.7	96.7	98.4	53.0
AG 2–4	Male	0.361 ± 0.019	0.963 (0.940–0.986)	Highest YI	0.382	89.7	89.6	99.4	30.6
				Pre-established*	0.370	100	73.5	100	16.1
				Cutoff >0.4	0.400	44.8	99.1	97.2	72.2
				50 % PPV	0.390	72.4	96.3	98.6	50.0
	Female	0.348 ± 0.017	0.919 (0.877–0.962)	Highest YI	0.361	88.2	82.0	99.2	21.0
				Pre-established*	0.370	67.6	92.5	98.1	32.9
				Cutoff >0.4	0.400	8.8	99.8	95.3	75.0
				50 % PPV	0.378	44.1	97.8	97.0	51.7
	Total	0.355 ± 0.019	0.955 (0.933–0.977)	Highest YI	0.370	93.7	84.0	99.6	23.6
				Cutoff >0.4	0.400	30.2	99.7	96.4	86.4
				50 % PPV	0.384	68.3	96.6	98.3	51.2
AG 5–8	Male	0.377 ± 0.023	0.941 (0.913–0.970)	Highest YI	0.396	92.5	83.6	99.5	22.6
				Pre-established*	0.370	100	42.4	100	8.2
				Cutoff >0.4	0.400	87.5	87.5	99.3	26.5
				50 % PPV	0.423	40.0	98.1	96.9	51.6
	Female	0.362 ± 0.023	0.967 (0.952–0.983)	Highest YI	0.384	93.3	89.8	99.6	32.1
				Pre-established*	0.370	100	71.5	100	15.4
				Cutoff >0.4	0.400	62.2	97.9	98.0	60.9
				50 % PPV	0.395	73.3	96.7	98.6	53.2
	Total	0.369 ± 0.024	0.958 (0.944–0.972)	Highest YI	0.395	89.5	90.3	99.4	32.5
				Pre-established*	0.370	100	57.8	100	11.0
				Cutoff >0.4	0.400	81.4	93.4	99.0	39.3
				50 % PPV	0.410	64.0	96.8	98.1	51.4

For both EI and FOHR, high NPV were achieved with the minimum NPV of all analyses of 95.7 % for EI in females of AG 2–4. However, sensitivity, specificity and PPV showed substantial variation depending on cutoff values, age and sex.

Exemplarily, for the widely accepted cut-off value of EI > 0.3 as an indicator for elevated ventricular volume, the following test criteria were obtained for the whole cohort: sensitivity 55.3%, specificity 97.1%, positive predictive value (PPV) 50.0%, negative predictive value (NPV) 97.6%. For the statistically best-performing cut-off value of EI > 0.279, by means of the highest YI, the test criteria were sensitivity 85.3%, specificity 85.1%, PPV 23.2%, NPV 99.1%. In males of AG 2–8, the PPV dropped to 18.1 % at the best performing cutoff value according to YI (EI > 0.280), and it reached only 38.7 % at EI > 0.3. A PPV of 51.6 % was reached at EI > 0.311. In females of AG 2–8, PPV was 25.1 % at the best performing cutoff value (EI > 0.273), and it was 55.3 % at EI > 0.3, however with a sensitivity of only 33.3 %, whereas sensitivity reached 43.6 % when using EI > 0.295, where PPV was closest to 50 % with 50.7 %.

To find a reasonable cutoff for FOHR, we first determined test criteria for the cutoff value with the highest YI in the whole cohort, as well as a second one with a PPV > 50 %. FOHR > 0.382 showed the highest YI, test criteria were sensitivity 96.0 %, specificity 85.3%, PPV 25.7%, NPV 99.8%. A PPV of over 50 % was reached at a cutoff of FOHR > 0.4, test criteria were sensitivity 70.7%, specificity 96.7%, PPV 53.0%, NPV 98.4%. We also evaluated the established cutoff of FOHR > 0.37, that was originally developed in children [22]. FOHR > 0.37 represented the best-performing cutoff value in AG 2–4 for males and females combined, but with a PPV of only 23.6 %. In no subgroup PPV reached more than 32.9 % (females AG 2–4) using FOHR > 0.37.

**Fig. 3:**
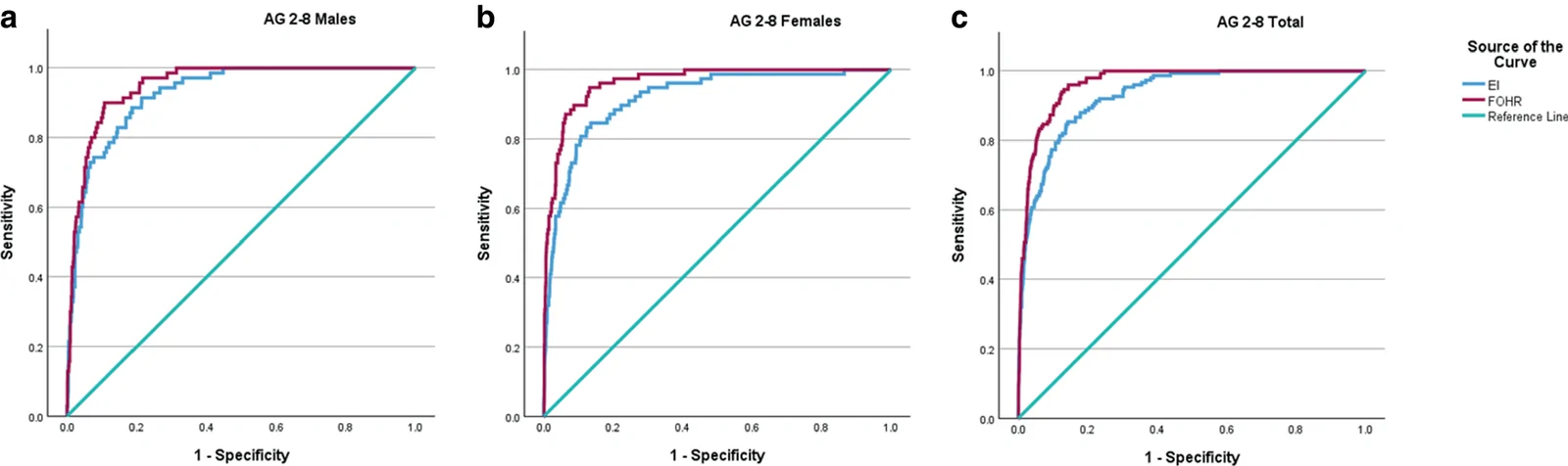
Receiver Operating Analysis of Evans’ Index (EI, blue curves) and Frontal and Occipital Horn Ratio (FOHR, red curves) for the diagnosis of lateral ventricle enlargement as determined by semiautomatic volumetry in (**a**) males of AG 2–8, (**b**) females of AG 2–8 and (**c**) both genders of AG 2–8. Areas under the Receiver Operating Curve (AUROC): AUROC_FOHR‑M_ = 0.951 vs. AUROC_EI‑M_ = 0.902, *p* = 0.012; AUROC_FOHR‑F_ = 0.964 vs. AUROC_EI‑F_ = 0.919, *p* < 0.001; AUROC_FOHR‑T_ = 0.964 vs. AUROC_EI‑T_ = 0.930, *p* < 0.001

**Fig. 4:**
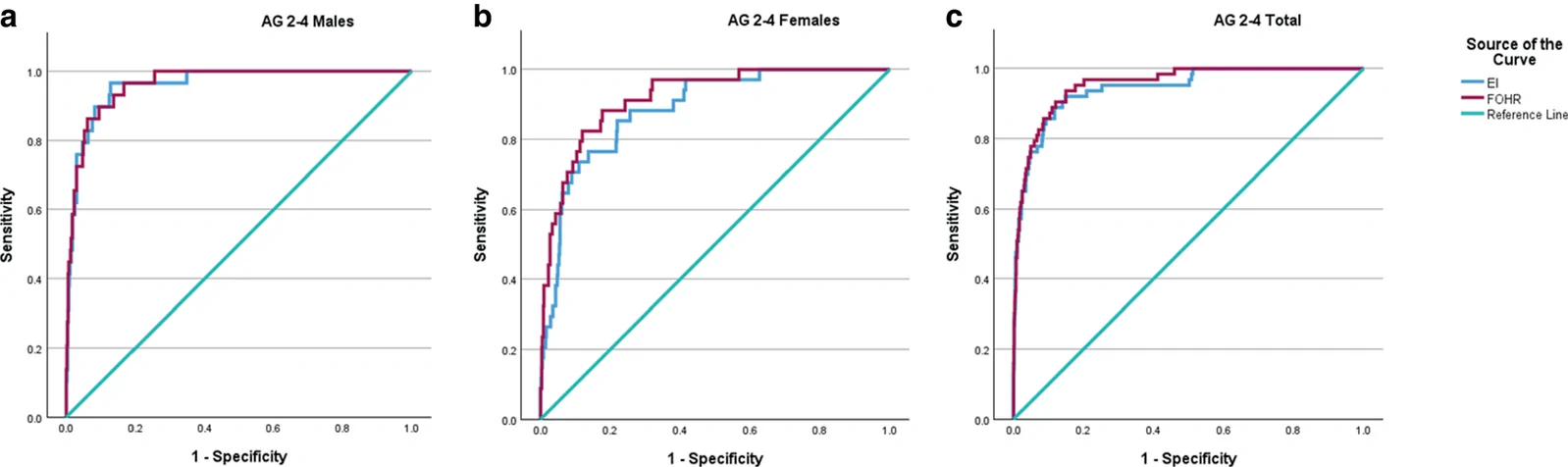
Receiver Operating Analysis of Evans’ Index (EI, blue curves) and Frontal and Occipital Horn Ratio (FOHR, red curves) for the diagnosis of lateral ventricle enlargement as determined by semiautomatic volumetry in (**a**) males of AG 2–4, (**b**) females of AG 2–4 and (**c**) both genders of AG 2–4. Areas under the Receiver Operating Curve (AUROC): AUROC_FOHR‑M_ = 0.926 vs. AUROC_EI‑M_ = 0.922, *p* = 0.813; AUROC_FOHR‑F_ = 0.919 vs. AUROC_EI‑F_ = 0.887, *p* = 0.128; AUROC_FOHR‑T_ = 0.955 vs. AUROC_EI‑T_ = 0.944, *p* = 0.266

**Fig. 5:**
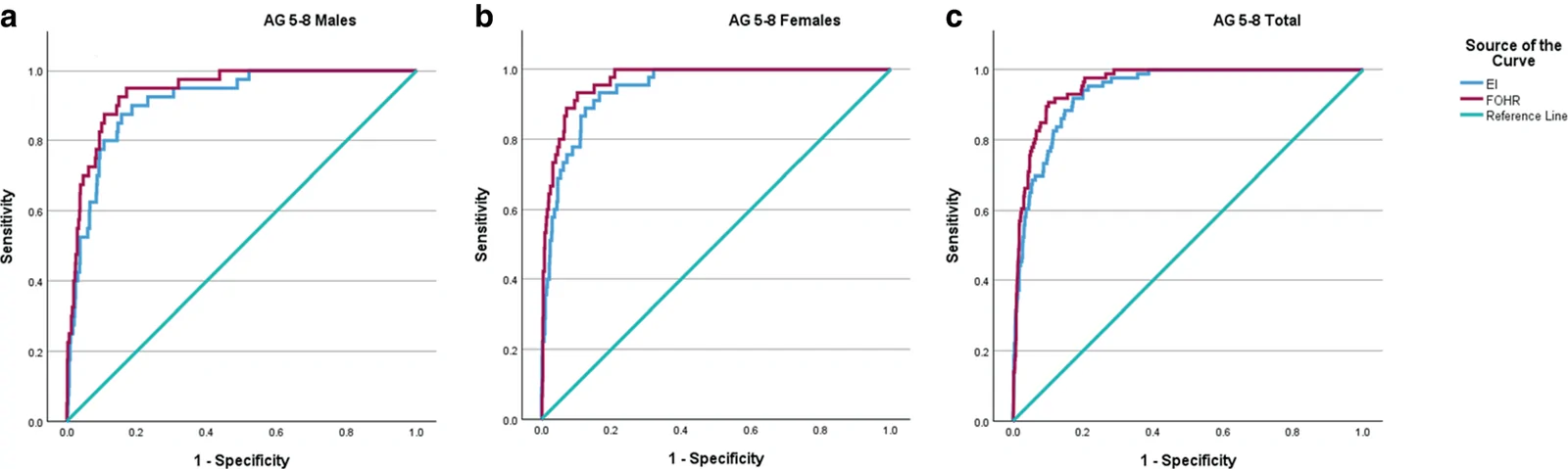
Receiver Operating Analysis of Evans’ Index (EI, blue curves) and Frontal and Occipital Horn Ratio (FOHR, red curves) for the diagnosis of lateral ventricle enlargement as determined by semiautomatic volumetry in (**a**) males of AG 5–8, (**b**) females of AG 5–8 and (**c**) both genders of AG 5–8. Areas under the Receiver Operating Curve (AUROC): AUROC_FOHR‑M_ = 0.941 vs. AUROC_EI‑M_ = 0.914, *p* = 0.052; AUROC_FOHR‑F_ = 0.967 vs. AUROC_EI‑F_ = 0.944, *p* = 0.012; AUROC_FOHR‑T_ = 0.958 vs. AUROC_EI‑T_ = 0.939, *p* = 0.006

For comparison of EI and FOHR, ROC analyses were carried out. The results are presented for the complete cohort (AG 2–8) in Fig. 3, for subgroups AG 2–4 in Fig. 4 and for subgroups AG 5–8 in Fig. 5, including statistical comparison between the AUROCs of EI and FOHR by means of DeLong-Test. Areas under the ROC (AUROC) with 95 % CI can be found in Table 2 for EI and in Table 3 for FOHR. For the complete cohort, FOHR performed significantly better compared to EI represented by an AUROC_FOHR_ of 0.964 (95%CI 0.955–0.973) vs. AUROC_EI_ 0.930 (95%CI 0.912–0.947), (z = 4.585, *p* < 0.001; DeLong test). This was also the case for males (z = 2.524, *p* = 0.012) and females (z = 3.805, *p* < 0.001) for AG 2–8. For AG 2–4 however, performance of EI and FOHR did not differ significantly (AG 2–4 total: z = 1.112, *p* = 0.266; AG 2–4 males: z = 0.235, *p* = 0.814; AG 2–4 females: z = 1.522, *p* = 0.128). For AG 5–8 FOHR performed better than EI for males and females combined (z = 2.741, *p* = 0.006) and for females (z = 2.517, *p* = 0.012). For males of AG 5–8 the level of significance was close to *p* < 0.05 with *p* = 0.052 (z = 1.941). When looking at the numbers for AG 8 (*n* = 37 total) only one out of 21 males and only one out of 16 women achieved LVV > 95th percentile of their respective age group and only one of 37 individuals’ LVV lay outside of the 95th percentile when both genders were combined. At the same time, there is substantial overlap of the mean ± SD of LVV (41.10 ± 10.98 ml) of AG 8 to the 95th percentile value for AG 5–8 (49.84 ml). Therefore, an additional subgroup ROC analysis was carried out with AG 5–7 only (not visualized) to avoid outliers in AG 8, revealing again superior test performance of FOHR vs. EI for males (z = 2.260, *p* = 0.024), for females (z = 2.947, *p* = 0.003) and for both sexes combined (z = 2.433, *p* = 0.015).

## Discussion

Evaluation of ventricular volume is routinely performed in everyday neuroimaging, either by simply eyeballing or ideally by using surrogate measurements for rule out or quantifying of ventricular enlargement. Pathological changes of the ventricular system often represent early manifestations of neurological diseases like hydrocephalus [40–43], dementia [44], multiple sclerosis [45] or in psychiatric illness [46–48]. Therefore, valid and technically robust, as well as quick ways to measure ventricular volume are of high interest, and several methods have been proposed [15, 16, 22, 23]. We present the first study that evaluates two of the most widely used methods by means of population-based data of Germany with a high number of probands and we present age-group- and sex-specific data on test performance.

To evaluate predictive values of the indices EI and FOHR in a sample of normal population, a volume-threshold for pathologically enlarged lateral ventricular volumes (LVV) had to be determined within the study cohort, because to our knowledge there are no universally accepted thresholds defining enlargement of lateral ventricles. This was done by statistical analysis, as others have done [5]. As the distribution of the semiautomatically derived LVV values was positively skewed and we wanted to carry out subgroup analyses with inevitably smaller sample sizes, especially being below *n* = 30 in AG 8 (*n* = 21 males; *n* = 16 females), we chose volume thresholds at the 95th percentiles to define elevated LVV in our cohort. This contrasts with the approach of a commercially available fully automatic segmentation software that uses mean plus two standard deviations to define the thresholds between normal and abnormal volumes [49], possibly relying on the central limit theorem, that we avoided to allow analysis of small subgroups. Of note, Ambarki et al. [5] used volumetry of the whole ventricular system of healthy elderly individuals, while we chose to use the volume of the lateral ventricles only, because that is the target of the manual measurements performed. For the whole cohort the determined LVV threshold was 45.26 ml, but for the subgroup analyses, the respective age- and sex-specific volume thresholds according to Table 2 were used. It is important to note that individuals older than 80 years old, representing only 1.2 % of the total sample, had a mean LVV of 44.10 ± 10.98 ml (age-group specific 95th percentile threshold 61.16 ml), which is very close to the threshold for abnormal LVV enlargement of the whole sample. This illustrates that careful consideration is necessary regarding volume threshold values. Therefore, we additionally provide age-group- and gender-specific thresholds for subgroup analyses.

Choosing the correct axial imaging slices is critical for correct measurements to determine EI and FOHR. Exemplarily, Toma et al. evaluated EI of 10 patients with clinically proven NPH and purposely did the measurements in 5 different axial positions for each individual, resulting in significant intraindividual variability [6]. Of three MRI studies evaluating EI in healthy adults, only one had a sample size of over 100, and all had very limited age groups. Astonishingly, regarding measurements, Inatomi et al. do not even provide details of how measurements were obtained [50], and Ishii et al. [51] as well as Ambarki et al. [5] did all their measurements in the same single axial slice, which is in contrast to the method as originally described. In the present study, measurements were done in a standardized manner [15, 52–54] in strictly axial slices, manually choosing up to two slices for EI or three slices for FOHR, with the maximum values for each parameter measured, as described above. We did not record the time needed to manually measure the diameters nor whether more than one measurement was necessary to find the maximum value for each individual diameter. However, diameter measurements are usually swiftly executed in axial brain MRI stacks, and adding one more diameter for FOHR seems unlikely to add more than a few seconds to the evaluation time, when the two measurements for EI have already been done. As the calculations of EI and FOHR were done automatically through spreadsheet formulas, we cannot estimate the duration it would take to obtain both ratios in a real-life setting. Using this standardized approach, we achieved good to excellent IRR, indicating these are valuable tools that generate reproducible results for day-to-day clinical evaluation.

The Evans’ Index (EI) represents the oldest index for quantification of the width of the ventricular system. Historically, it had been established for pneumoencephalography [15, 16], but in the meantime has also been validated for MRI and CT [52–54], with an EI > 0.3 regarded as indicator for an enlarged ventricular system [17]. The measurements and arithmetic needed to calculate EI are simple and quickly executed, which is underlined by excellent IRR-Values that are in line with the results of other groups [55]. In the present study we found a mean EI of 0.257 ± 0.025 within the total sample of normal population, with age-group- and sex-specific differences. Differences in the correlation coefficients for EI and LVV between AG 2–4 and AG 5–8, albeit significant, were rather small compared to the differences between age and LVV. When considering the whole sample 166 probands (5.6 %) achieved an EI > 0.3, and of those, all but 6 showed actual LVV above mean values for their respective age groups. In conclusion, EI seems to be a robust parameter relatively independent of an individual’s age. In a Japanese study on the prevalence of NPH, Tanaka et al. found an EI > 0.3 in 7.0 % [56]. The Japanese cohort included only probands older than 65 years of age, which likely explains the different percentage of individuals with enlarged ventricular volumes, given that in the present study EI also increased with age. This is nicely reflected in our data, as in AG 5–8, including only participants older than 50 years of age, we found an EI > 0.3 in 7.5 % of cases.

For interpreting test performance, our data allows for a differentiated approach. Exemplarily, for the whole sample, an EI > 0.279 represents the best performing cutoff value for ventricular enlargement, regarding sensitivity and specificity as determined by YI, and yields a high NPV of 99.1 %. However, the PPV is low (23.2 %) compared to a PPV of 50.0 % for the accepted cutoff value of EI > 0.3. Therefore, the cost of higher sensitivity would be a false positive rate of ¾ of patients, which does not seem feasible. The NPV of EI > 0.3 is still excellent with 97.1%, but sensitivity is considerably lower (55.3 %). Comparable relations are found between best-performing cutoff-values as determined by YI and the pre-established cutoff of EI > 0.3 in all age- and sex-specific subgroup analyses. In cases where the cutoff of EI > 0.3 did not reach—or was not the closest to—a PPV of 50 %, an additional cutoff with PPV ≥ 50 % was evaluated. The difference between the latter and EI > 0.3 was smaller than between the best-performing cutoff and EI > 0.3 in all analyses.

In conclusion, although the best-performing EI cutoff values, either for the whole sample or age- and sex-specific, may be used to safely rule out ventricular enlargement, the pre-established cutoff of EI > 0.3 has the advantage of a lower rate of false positives. In some scenarios an additional cutoff performs better than EI > 0.3 to reduce false positive rates. We must keep in mind that even the more conservative cutoff values carry the risk of false positive diagnosis in nearly half of our patients, and at the expense of a lower sensitivity, therefore, we should be cautious to rely on EI alone, if elevated. This is especially important when dealing with patients without symptoms and without a specific clinical question regarding the ventricular system, when reporting enlargement of the ventricles as a side diagnosis with uncertain clinical significance bears the risk of unnecessary further workup.

The “Frontal and Occipital Horn Ratio” (FOHR) was developed in 1998 by O’Hayon et al., to factor in asymmetric ventricular enlargement, initially in pediatric patients [22]. Since then, it is also sometimes used in adult patients [57], regardless of missing validation. We present the first data for FOHR in a large sample of adult normal population in Germany. The determined mean values of FOHR = 0.363 ± 0.024 in our cohort are close to the originally published values of 0.37 ± 0.026 in children. In our cohort FOHR showed continuous progress markedly from AG 5 to AG 8. FOHR showed strong correlation with LVV (r_s_ = 0.741; *p* < 0.001), more pronounced in higher age groups AG 5–8 (r_s_ = 0.759; *p* < 0.001).

Using O’Hayon‘s cutoff value of FOHR > 0.37 [22], sensitivity and specificity in our complete sample were 100 %, and 69.0%, respectively, with a NPV of 100 % and a PPV of only 14.6%. NPV was high (96.4 to 100 %), but PPV was low (8.2 to 32.9) in all age- and sex-specific subgroup analyses carried out, with a PPV of only 11.0 % for AG 5–8 using the cutoff of FOHR > 0.37. Therefore, the cutoff value of FOHR > 0.37, originally validated in children, seems questionable in the adult population. Regarding the complete cohort, the highest combination of sensitivity and specificity as determined by YI is found for a FOHR > 0.382 (96.0 %, resp. 85.3%), however with a PPV of only 30.7%. A PPV > 50 % is found for FOHR > 0.4, with sensitivity of 70.7% and specificity of 96.7% (PPV = 53.0%). The cutoff of FOHR > 0.4 was therefore included in the subsequent subgroup analyses for FOHR, in addition to the best-performing cutoff (YI), the pre-established cutoff and the cutoff resulting in a PPV of ≥ 50 %. Interestingly, in AG 2–4 when both genders were combined, the best-performing cutoff was equal to the pre-established cutoff of FOHR > 0.37, however with a low PPV of 23.6 %; a PPV of 51.2 % was reached at FOHR > 0.384 in this subgroup, which lies closer to the pre-established 0.370 than to 0.400. The age- and sex-specific range of cutoff values for FOHR is more complex compared to EI. A possible reason being that correlation between FOHR and age was stronger compared to EI (r_s_ = 0.409 vs. r_s_ = 0.117; *p* < 0,001), with significant differences between male and female probands for FOHR (0.356 ± 0.022 vs. 0.371 ± 0.023; *p* < 0.001). While the cutoff of FOHR > 0.4, derived from the whole sample, seems to yield acceptable performance at first glance, it does not perform as well in all subgroup analyses. Therefore, FOHR > 0.4 may be used to rule out ventricular enlargement in the adult population, however better performing cutoff values are available depending on specific age-groups and gender, that may be applied especially when the initial result is close to the cutoff.

Comparison of AUROC revealed no significant differences between the test performance of FOHR and EI in AG 2–4. Therefore, in patients below 50 years of age, FOHR does not usually add confidence to the results of EI in the diagnosis of ventricular enlargement. Cases with asymmetric ventricular enlargement—the purpose FOHR was initially developed for—pose a possible exception to this observation, however, we cannot verify this with our data. We propose that extra effort for the additional measurement of the occipital horn diameter and the application of the more complex arithmetic should be preserved for obvious asymmetric enlargement of the lateral ventricles, especially when a seemingly normal EI value does not reflect the radiologist’s impression of the situation.

For AG 5–8 AUROC comparison revealed significantly superior performance of FOHR over EI. In conclusion, FOHR performs better than EI in older probands, and it performs better in older than in younger individuals, when the correct cutoff is used, contradicting results of Ambarki et al. [5]. We therefore suggest using FOHR as an additional parameter to confirm borderline or elevated EI-values in individuals ≥ 50 years of age.

## Limitations

An important limitation lies in the source of the data, including a representative sample of adult normal population of northeastern Germany, exclusively. Therefore, although presenting to our knowledge an evaluation of EI and FOHR in the largest population-based sample so far, generalization to other populations is limited, and validation in other population-based samples would be desirable. Furthermore, the diagnosis of ventricular enlargement in this study was derived merely by statistical analysis of the ventricular volumes within the sample rather than from predetermined clinical diagnosis of hydrocephalus. However, because such information was not available, and the sample size exceeds both in total as well as in the majority of subgroups previously published data on the topic using a comparable definition of ventricular enlargement, we are positive that this is a valid approach. Limited evidence is available for the age group of 80 years and older, because of substantial overlap of ventricular size thresholds as well as EI and FOHR ranges with even the most conservative cutoff values. Owing to the small size of this subgroup, isolated comparison of the AG 8 to other individual AGs was not feasible. Apart from subjects excluded from the study for evident brain pathology like space occupying tumors or defects caused by ischemic stroke or hemorrhage, there was no further information, nor evaluation of brain pathology as possible cause for changes in ventricular volumes. No information on brain function or gray-white-matter distribution was available for statistical analysis. In the dataset, there was also no information to correlate whether those individuals with elevated ventricular volumes as determined by our definition of > 95th percentile suffered from clinically apparent, i.e. symptomatic hydrocephalus, or for example neurodegenerative diseases. Regarding manual measurements, no repeat measurements were performed by the raters, and we cannot determine intra-rater-reliability. In summary, EI and FOHR in this study are evaluated for their performance in detecting ventricular enlargement in the normal adult population in north-eastern Germany rather than symptomatic hydrocephalus in the global population, and careful interpretation of the parameters and their cutoff values is advised when applying them to patients with hydrocephalus and outside of Germany or at least Central Europe. Validation studies including different populations as well as patients with hydrocephalus would be helpful in the future.

## Conclusions

In the present study, EI and FOHR are validated in a reasonable sized representative sample of normal adult population of Germany for the first time.

In our opinion, a single “one fits all” cutoff value for any test has the advantage of being easy to memorize, which helps propagating it and facilitates application in everyday use, without having to first memorize or repeatedly consult complex tables, which is time-consuming. In the past, this has been assumed for the pre-established cutoff EI > 0.3, and sometimes even for FOHR > 0.37 although FOHR was not validated for the adult population. In the endeavor to keep it simple, we think that our data justifies using EI > 0.3 in the normal adult population in Germany, however, with three important caveats:

- the strength of EI lies in a high NPV, which is ideal to confidently rule out ventricular enlargement. Careful interpretation is advised for a positive test. Confirmatory measures may be necessary to verify enlargement, either by adjusting the cutoff for EI to age- and sex-specific values (e.g. by consulting Table 3), or by additionally applying FOHR, especially in individuals ≥ 50 years of age.
- EI does not account for asymmetrical enlargement of the occipital horns rather than the frontal horns. In this case FOHR should instead be used.
- Our data provides insufficient evidence for very old individuals over 80 years of age. EI was 0.281 ± 0.029 (mean ± SD) in this subgroup, overlapping even the most conservative cutoff of EI > 0.309 in AG 5–8. In this age group, other methods should be considered.

Similarly, for FOHR, a cutoff value of > 0.4 may be used for an initial evaluation of potential ventricular enlargement in the normal adult population in Germany, with important specific caveats:

- Application of FOHR in adult individuals < 50 years of age should be avoided due to low sensitivity, and non-superiority to the simpler EI, unless there is suspicion for asymmetric enlargement of the occipital horns.
- When FOHR results are close to the cutoff value, additional consideration of the age- and sex-specific values seem useful (Table 4).
- Again, our data provides insufficient evidence for very old individuals over 80 years of age. FOHR was 0.400 ± 0.021 (mean ± SD) in this subgroup, overlapping even the most conservative evaluated cutoff of FOHR > 0.410 in AG 5–8. In this age group, other methods should be considered.

## Data Availability

The data that support the findings of this study are not openly available due to reasons of sensitivity and are available from the corresponding author upon reasonable request.
